# A Comparative Study of Hand-Sewn and Stapled Anastomosis in Gastrointestinal Surgeries

**DOI:** 10.7759/cureus.71264

**Published:** 2024-10-11

**Authors:** Vinayak V Kshirsagar, Himashree MP

**Affiliations:** 1 Department of General Surgery, Dr. D. Y. Patil Medical College, Hospital, and Research Centre, Dr. D. Y. Patil Vidyapeeth (Deemed to be University), Pune, IND

**Keywords:** complications after bowel anastomosis, efficiency of stapler vs hand sewn anastomosis, hand-sewn anastomosis, small bowel resection, stapled anastomosis

## Abstract

Background

Bowel anastomosis is a key part of gastrointestinal surgery where two sections of the intestines are connected. It is a critical step as it restores the digestive tract's continuity after removing damaged or diseased bowel. Stapler devices are a new method that helps connect bowel loops faster and with less tissue damage. This study aimed to evaluate and compare the safety and effectiveness of stapled anastomosis versus hand-sewn anastomosis in surgeries involving the bowel.

Methods

In this prospective non-randomized study, the study population included 60 patients with various gastrointestinal surgeries admitted to our institution from August 2022 to June 2024 within a single unit. The inclusion criteria included all patients aged over 18 years who underwent surgeries such as gastrojejunostomy, hemicolectomy, and small bowel resection with anastomosis. The exclusion criteria were age under 18, pregnancy, and immunocompromised states. Patients were divided into two groups based on how their digestive tract was reconstructed: 28 had stapled anastomoses and 32 had hand-sewn anastomoses, either as elective or emergency surgery. We examined multiple outcomes such as surgery duration in hours, time until bowel function returned post surgery, length of hospital stay in days, anastomotic leak rate, albumin levels to leak rate, post-surgery pain, and surgical site infection (SSI) rate for the two groups. Southampton wound grading system and the visual analog scale (VAS) were used to assess the SSI and pain score, respectively.

Results

When comparing the operation time for the suture and staple groups among the elective cases, the p-value was significant (0.02), with 15 patients having prolonged surgery in the suture group compared to only five in the staple group. Regarding the time until the return of bowel sounds post surgery, the p-value was significant (0.02) for elective cases in the two groups, with nine patients having their bowel sounds returning after the third postoperative day (POD) in the suture group. When comparing the duration of stay in the hospital for elective cases for the staple and suture groups, the p-value was significant (0.04), with eight patients staying after the seventh POD in the suture group. When comparing the leak rates for the two groups for elective cases, the p-value was significant (0.04), with four leaks in the suture group and no leaks in the staple group. On comparing the association between the albumin level and anastomotic leak, the p-value was significant at 0.001 and 0.0006 for the suture and staple groups, respectively, with more leaks associated with an albumin level of <3 mg/dl. When comparing pain scores of the suture and staple groups separately in elective and emergency settings, the p-values were both 0.4, which is not significant. Among the elective cases comparing SSIs for the suture and staple groups, the p-value was significant (0.04), with six patients having SSIs in the suture group compared to one patient in the staple group. Among the emergency cases, when comparing SSIs for the suture and staple groups, the p-value was not significant (0.1).

Conclusion

In elective surgeries, stapled anastomosis reduces surgery time, accelerates bowel function recovery, enables earlier discharge, and lowers anastomotic leak rates compared to sutures. The staples group showed no difference from sutures in the leak rate to albumin levels and pain while offering superior SSI prevention.

## Introduction

The anastomosis of bowel loops is among the most important aspects of surgical procedures performed on the gastrointestinal tract. A technical innovation that assists anastomosis of bowel loops with reduced tissue injury and a shorter duration is the stapler device. Generally, ensuring successful bowel anastomosis using staples or sutures requires precisely aligning the bowel edges without tension and ensuring they have sufficient blood supply while being sealed tightly against air and water.

After intestinal anastomosis, the early complication (which occurs by the end of the first week post surgery) [1] causing the most concern is anastomotic leakage, an outcome assessed in our study for the suture and staple groups in elective and emergency settings. The healing of an intestinal anastomosis can generally be divided into three stages [2] as follows: the inflammation phase, the fibroplasia phase, and the remodeling period. Around the second to third postoperative days (PODs), the inflammatory phase is followed by the fibroplasia phase; a delay in this transition from the inflammatory phase to the fibroplasia phase can be caused by any systemic or local condition. This delay might result in poor healing and anastomotic leaking [3]. Anaemia, diabetes mellitus, malnutrition with hypoalbuminemia, vitamin deficiencies, and steroid medication are all examples of systemic factors that can increase the likelihood of anastomotic leakage [4].

Currently, no standardized guidelines exist for choosing between stapler devices and traditional hand-sewn methods in bowel surgery [5]. Surgeons typically decide based on their experience and the specific conditions observed during surgery, such as the condition of the bowel and the nature of the pathology requiring resection and anastomosis. We conducted a prospective observational study to gather information regarding the traditional hand-sewn versus the stapled anastomosis and determine which method of bowel anastomosis was the best for the patient based on our single-center analysis. We examined multiple outcomes such as surgery duration in hours, time until bowel function returned post-surgery, length of hospital stay in days, anastomotic leak rate, albumin levels to leak rate, post surgery pain, and the surgical site infection rate (SSI) for the suture and staple groups in elective and emergency settings. Southampton wound grading system and the visual analog scale (VAS) were used to assess the SSI and pain score, respectively, for the two groups.

## Materials and methods

Patient selection

The study population included 60 patients with various gastrointestinal diseases admitted to our institution, Dr. D. Y. Patil Medical College, Hospital, and Research Centre, Dr. D. Y. Patil Vidyapeeth (Deemed to be University), Pune, India, from August 2022 to June 2024 within a single unit. The inclusion criteria included all patients aged over 18 who underwent surgeries such as gastrojejunostomy, hemicolectomy, and small bowel resection with anastomosis. The exclusion criteria were patients aged under 18 years, pregnant, and with immunocompromised states. The study covered elective and emergency surgeries where the patients underwent laparotomy to manage their bowel pathology. Before participating, all patients gave written consent after receiving detailed information about the study. The patients were divided into two groups based on how their digestive tract was reconstructed: 28 had been stapled and 32 had hand-sewn anastomosis, either as elective or emergency surgery. Our hospital’s Institutional Review Board (IRB) (IRB approval name: Research and Recognition Committee under the Faculty of Medicine) approved the study (research protocol number: IESC/PGS/2022/87), which followed ethical guidelines in the Declaration of Helsinki and adhered to good clinical practices and local laws.

Data were collected prospectively until patients were discharged from the hospital after surgery. We recorded information about their recovery by comparing various clinical outcomes post surgery for patients who underwent stapled and hand-sewn bowel anastomosis. The parameters assessed included the duration of surgery (in hours), time to return of bowel function (determined by auscultation of bowel sounds), length of hospital stay (in days), anastomotic leak rates, and serum albumin levels to anastomotic leak rates in both groups. Additionally, we compared postoperative pain and the rate of SSIs for the two groups. All these outcomes were assessed separately in elective and emergency surgery settings. The Southampton wound grading system was used to assess SSI, while the VAS was employed to measure pain scores.

Study design

Our study is a prospective observational study (non-randomized) comparing multiple outcome variables, as mentioned above, for two surgical groups, the suture and staple groups. Based on similar studies in gastrointestinal surgeries, the mean and standard deviation (SD) for hand-sewn and stapled techniques for the duration of surgery were used for sample size calculation [6-8]. Hence, considering the mean and SD as 2.71 ± 0.393 and 1.93 ± 0.56, respectively, with a 95% confidence level and a 5% significance level, we calculated a sample size of 34 using WINPEPI 11.38 (developed by Abramson JH, Jerusalem, Israel; http://www.brixtonhealth.com/pepi4windows.html). As our study progressed, we enrolled more patients than initially planned, which has strengthened the reliability and impact of our findings. The collected data in our observation are interpreted using OpenEpi (replica) of EpiInfo software released by the Center for Disease Control (CDC) in Atlanta, GA, to statistically determine significant associations between the two groups. The calculations were categorized and analyzed using the chi-square test in this study to find a test value and its significance for the two groups.

Overview of the operative techniques

**Figure 1:**
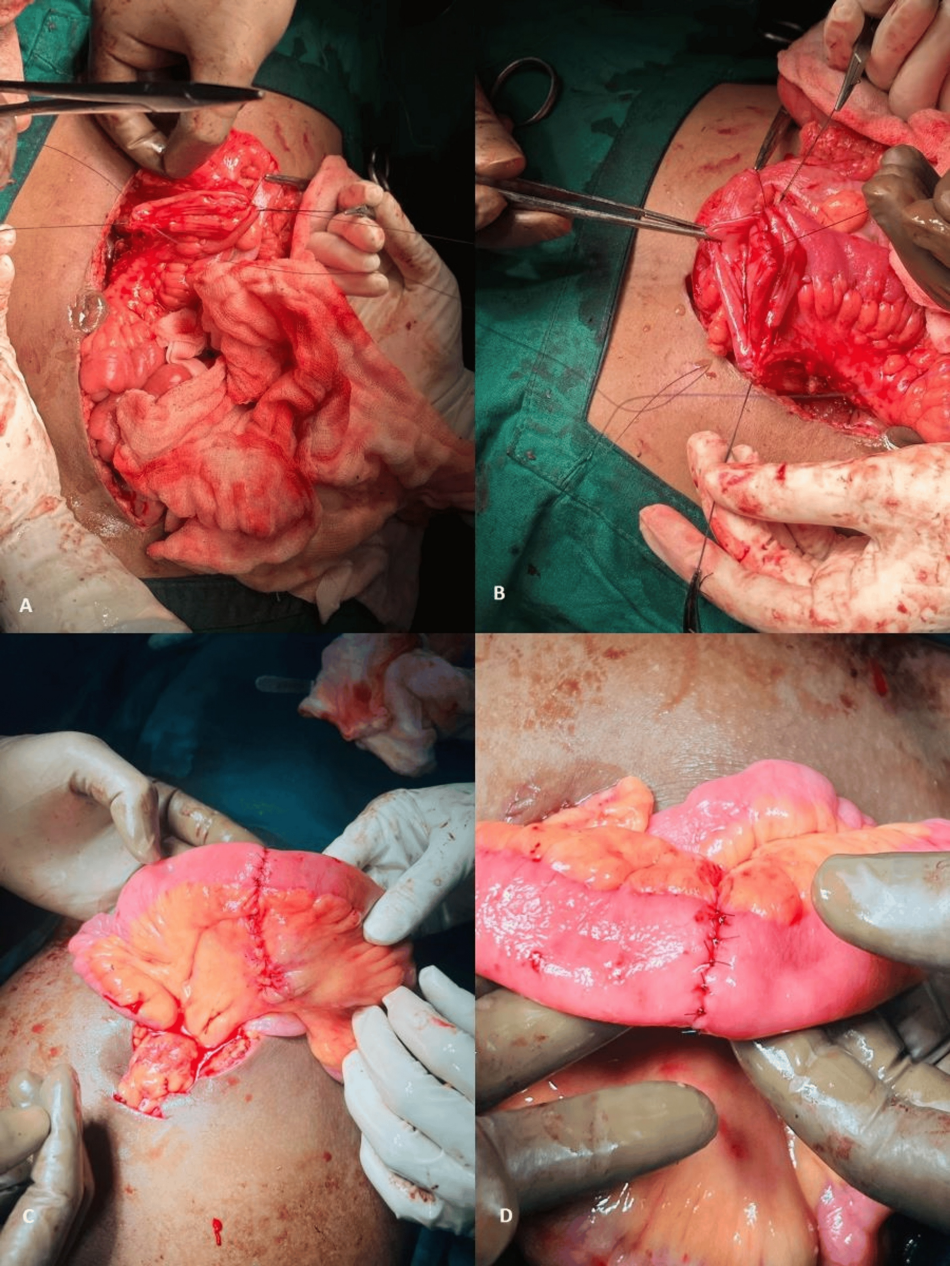
Hand-sewn anastomosis after small bowel segmental resection in one of our patients (end-to-end method) Panes A–D depict the start of anastomosis along the posterior layers of two bowel ends to the completion of the anterior layers of the two bowel ends.

**Figure 2:**
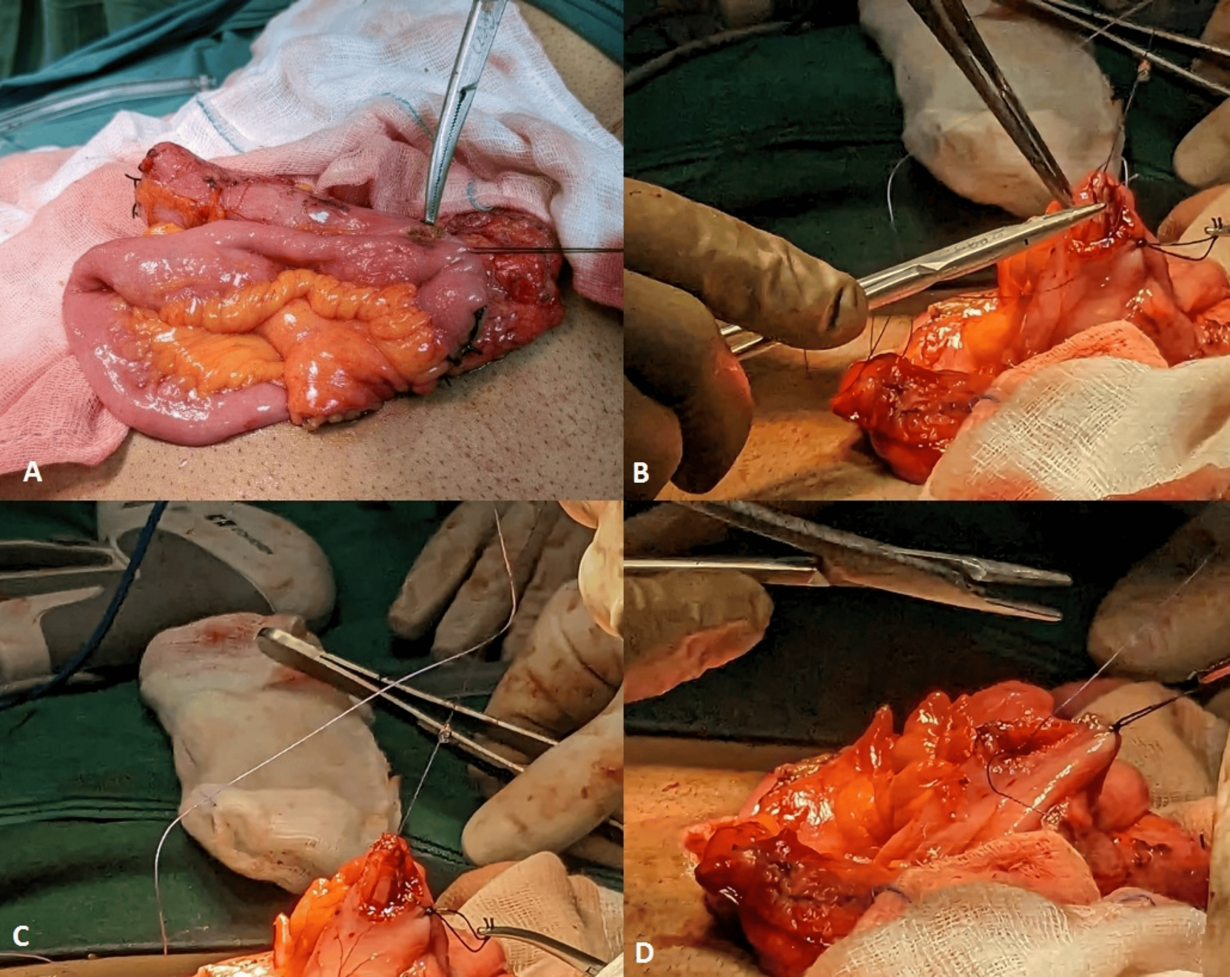
Panes A–D depict enterotomy closure after stapled anastomosis in one of our patients; Pane A shows the enterotomy, Panes B and C show the start of enterotomy closure from inside (luminal aspect), and Pane D shows the closure of the outer layer of the enterotomy.

The study included surgeries such as open gastrojejunostomy, open right and left hemicolectomy, and open small bowel resection with anastomosis. For hand-sewn anastomosis, the sutures used include 3-0 polydioxanone suture (PDS), 3-0 silk, and 3-0 polyglactin (Vicryl). The suture anastomosis was performed using the anatomical end-to-end method for small bowel and larger bowel resections, or end-to-side for ileocolic anastomosis and side-to-side for gastrojejunostomy, using two layers on posterior and anterior aspects; the first layer was full-thickness continuous, and the second layer was the interrupted seromuscular reinforcing layer (Figure 1A-1D). Vicryl sutures were used for the inner layer and silk sutures for the outer layer. The staplers used in the procedures were the linear cutter stapler and endoscopic gastrointestinal anastomosis (endo GIA) triple-layer stapler. The linear cutter stapler was used to resect the bowel and the endo GIA for anastomosis (Video 1). Most of the anastomoses were performed using an end-to-end functional procedure after creating a small enterotomy for stapler limb insertion; the enterotomy was closed in two layers with Vicryl and silk sutures (Figure 2A-2D) [9]. Only in two elective cases, we used the Barcelona technique of two-stapling anastomosis [10] using a linear cutter stapler, which is also a variant of end-to-end functional anastomosis.

**Video 1:** Stapled anastomosis after small bowel resection conducted in one of our patients (functional end-to-end method)

## Results

**Figure 3:**
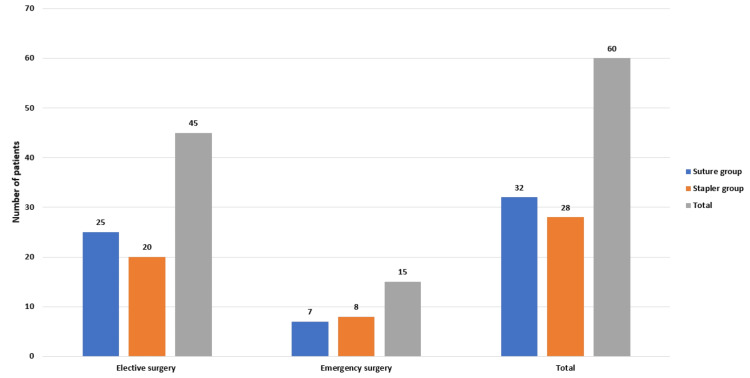
The distribution of cases among the suture and staple groups in elective and emergency settings

In our study, of the total 60 patients, 45 were elective cases, with 25 in the suture group and 20 in the staple group. Out of the 60 patients, 15 were emergency cases, with seven in the suture group and eight in the staple group. Thus, 32 patients were in the suture group and 28 in the staple group (Figure 3).

**Figure 4:**
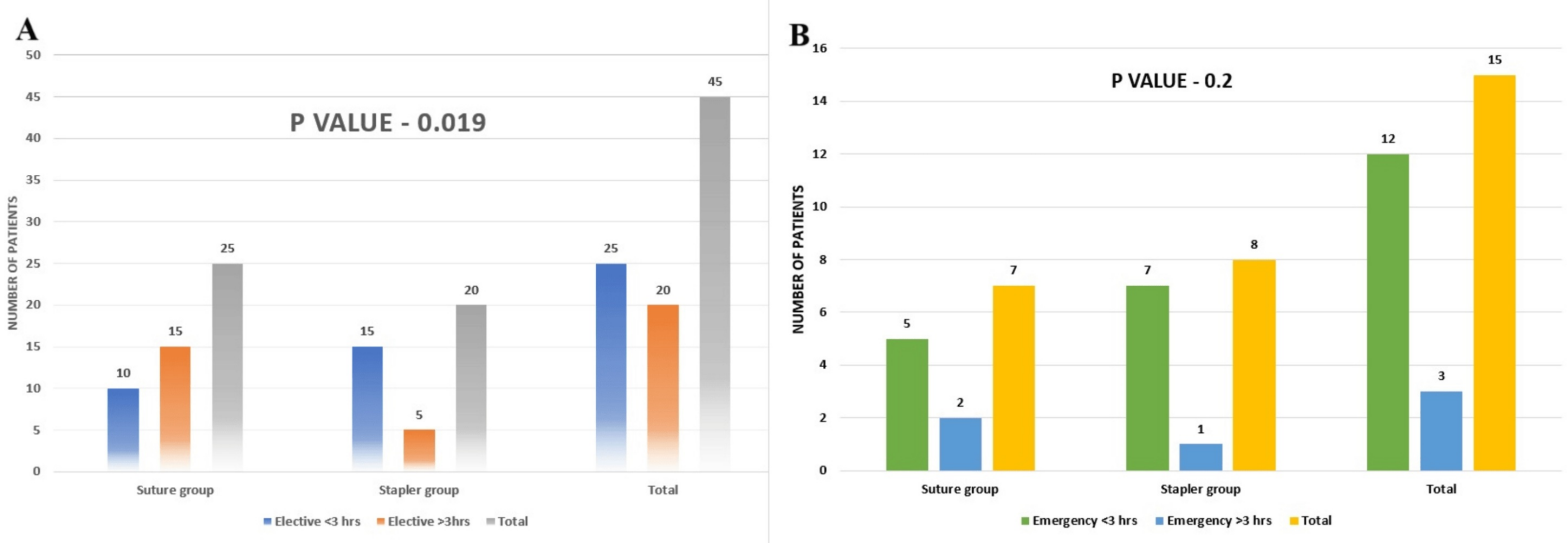
Panes A and B depict bar charts of duration of surgery for the two groups among elective and emergency cases, respectively hrs: hours

Initially, we compared the durations of surgery for the two groups in elective cases. To simplify our analysis using the chi-square method, we categorized the surgery duration as either less than three hours or more than three hours. We found that using staplers significantly reduced the surgery time in elective cases (p-value 0.02) (Figure 4A), indicating a productive outcome. However, this reduction in surgery time was not observed in emergency cases, where the p-value was 0.2 (Figure 4B).

**Figure 5:**
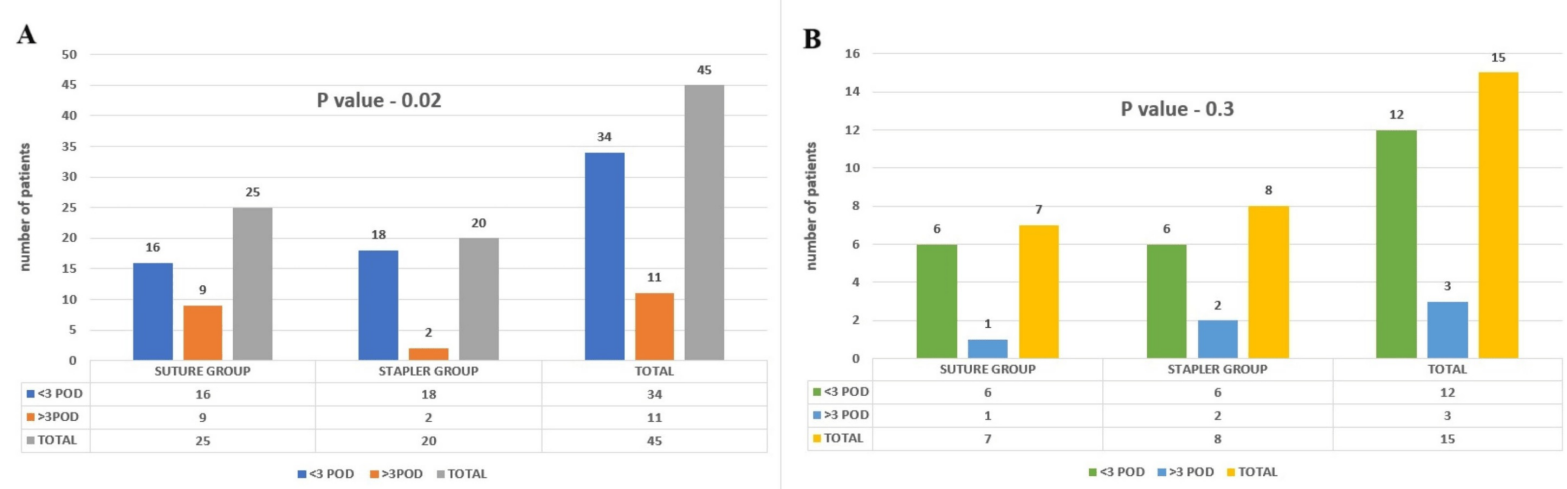
Panes A and B show bar charts of time of return of bowel sounds for the two groups among elective and emergency cases, respectively POD: postoperative day

Following our initial analysis, we examined the duration after which bowel sounds resumed in patients who underwent either suture or staple procedures in elective and emergency contexts. To facilitate our comparison, we segmented this period into categories: two to three days (<3 days) and more than three days (>3 days), using these distinctions to conduct a chi-square test to compare the two groups. Out of 20 patients who received stapled anastomosis, 18 experienced early return. Conversely, in the suture group of 25 cases, only 16 had bowel sounds returning within three days, with nine taking longer than the fourth POD. Our findings revealed that the application of staplers in elective surgeries significantly expedited the restoration of gastrointestinal function, leading to an earlier return of bowel sounds, with a statistically significant p-value of 0.04 (Figure 5A). This advantage for the staple group was not observed in emergency cases (p-value 0.3). However, out of 15 emergency patients, both groups had six patients with bowel sounds returning within the second to third POD (Figure 5B).

**Figure 6:**
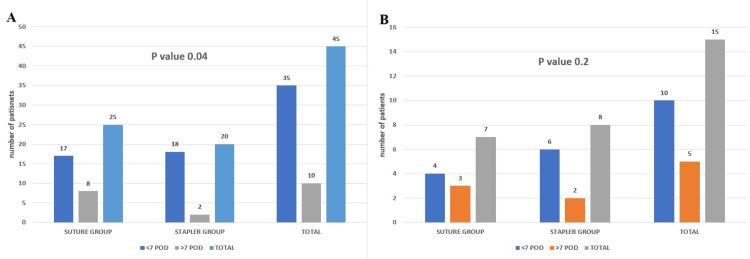
Pane A depicts a bar chart for duration of stay (in days) in hospital for the two groups for elective cases, and pane B depicts a bar chart for duration of stay (in days) in hospital for the two groups for emergency cases. POD: postoperative day

Continuing from our earlier analyses, we investigated how long patients stayed in the hospital after surgery in elective and emergency cases. We categorized patients based on whether they were discharged within seven days or after more than seven days post surgery. After analyzing the data and performing the calculations, we discovered that for elective cases, patients in the staple group were significantly more likely to be discharged earlier (p-value 0.04) (Figure 6A). However, in emergency surgeries, using a stapler did not result in faster discharge from the hospital (p-value 0.2) (Figure 6B).

**Figure 7:**
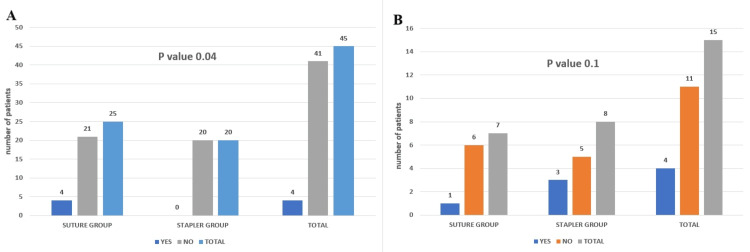
Anastomosis leak between two groups in elective cases (A) and emergency cases (B)

In our analysis of anastomotic leaks, a critical concern following gastrointestinal surgeries, we relied on clinical and radiological criteria such as localized fluid accumulation near the anastomosis, elevated body temperature, and alterations in vital signs for diagnosis. Of the 45 elective cases studied, 25 patients underwent suturing procedures, and approximately four experienced leaks. Conversely, none of the 20 cases treated with staples exhibited leaks. These findings indicate that the use of staples was associated with a lower incidence of leaks compared to suturing techniques in elective surgeries, supported by a statistically significant p-value of 0.04 (Figure 7A). However, this advantage was not observed in emergency surgeries, where suturing and stapling methods resulted in similar leak rates, as reflected by a p-value of 0.1 (Figure 7B).

**Figure 8:**
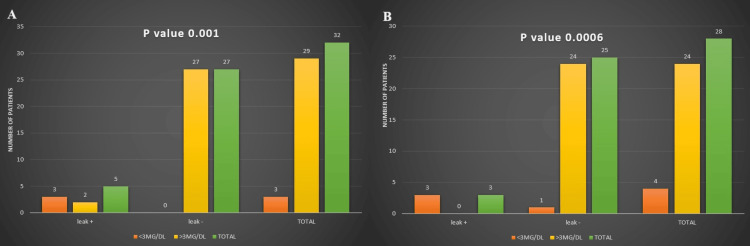
Comparing the leak rate to albumin leve Panes A and B depict an anastomotic leak in patients in the suture and staple groups, respectively, in comparison to an albumin level below or above 3 mg/dl.

Subsequently, our analysis investigated the association between albumin levels and the incidence of anastomotic leaks across elective and emergency surgical contexts. We classified patients based on their albumin levels: those with less than 3 mg/dL were categorized as having low albumin, while those with more than 3 mg/dL were considered to have high albumin. Using chi-square tests, we discovered a statistically significant association between low albumin levels and a higher occurrence of anastomotic leaks in elective and emergency scenarios (p-value 0.001 and 0.0006, respectively) irrespective of staple or suture use (Figures 8A-8B).

**Figure 9:**
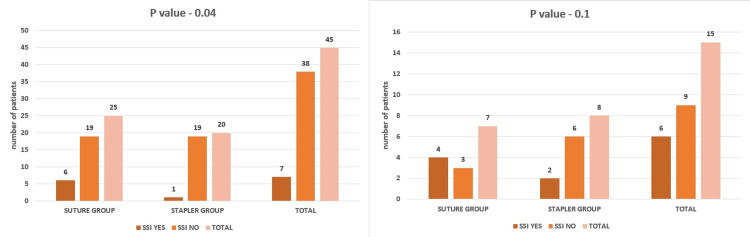
Comparing surgical site infections for the two groups in elective (A) and emergency (B) cases

To evaluate surgical wounds, we used the Southampton wound grading score (Table 1) [11], where a score above two indicates a positive finding for SSI. When comparing SSI rates among the two groups, we found a distinct difference in outcomes based on the choice of suture or stapled anastomosis. Specifically, in elective surgeries, patients with stapled anastomosis had a significantly lower incidence of SSI compared to those who underwent suture anastomosis (p-value 0.04) (Figure 9A).

**Table 1:** Southampton wound grading system Source: [11]

Grade	Appearance
0	Normal healing
I	Normal healing with mild bruising or erythema
Ia	Some bruising
Ib	Considerable bruising
Ic	Mild erythema
II	Erythema plus other signs of inflammation
IIa	At one point
IIb	Around sutures
IIc	Along wound
IId	Around wound
III	Clear or hemoserous discharge
IIIa	At one point only (=2cm)
IIIb	Along wound(>2cm)
IIIc	Large volume
IIId	Prolonged (>3days)
IV	Pus
IVa	At one point only (=2cm)
IVb	Along wound (>2cm)
V	Deep or severe wound infection with or without tissue breakdown; hematoma requiring aspiration

Conversely, in emergency cases, the advantage of using staples did not translate into a lower likelihood of SSI compared to suture anastomosis (p-value 0.2) (Figure 9B). This indicates that staples may offer benefits in terms of reducing the SSI risk in elective settings.

**Figure 10:**
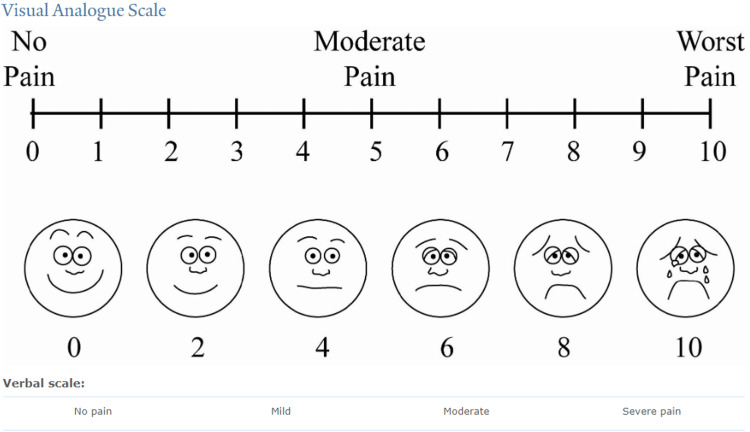
Visual analog scale Source: [12]

**Figure 11:**
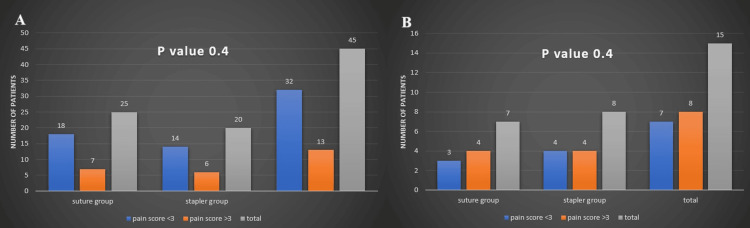
Comparing pain scores for the two groups in elective (A) and emergency (B) cases

Finally, we compared the levels of postoperative pain for the two groups in elective and emergency settings. We used a VAS ranging from one to 10 with smiley faces to assess subjective pain (Figure 10) [12], considering any score above three during the first three days after surgery as significant pain. Our analysis showed no statistically significant difference in postoperative pain between the groups in either elective or emergency surgeries, with p-values of 0.4 for both scenarios (Figures 11A-11B). This indicates that whether suturing or stapling techniques were used did not significantly affect the amount of pain experienced after surgery, whether it was planned or performed as an emergency. These results underscore the importance of managing postoperative pain effectively using standardized protocols and interventions, regardless of the surgical method employed.

## Discussion

Comparing the durations of surgery for the suture and staple groups

In our study of 60 patients, 45 underwent elective surgeries, with 25 in the suture group and 20 in the staple group. When we compared the durations of surgery for these groups, we found that surgeries using staples were completed faster than those using sutures. This difference was statistically significant. This could be because in elective cases, where the surgical planes are clear and fewer complications occur during the initial stages of dissection and resection, the time needed for the anastomosis becomes the main factor determining the overall surgery duration between the two techniques. Using staples enables faster completion of surgery compared to hand-sewn techniques, which typically take 30 to 45 minutes, depending on the surgeon’s experience [6,13].

In emergency surgeries, using staples for anastomosis does not significantly affect the overall duration of the surgery compared to sutures. This is probably because the total surgery time is primarily influenced by the underlying condition necessitating the emergency surgery. Conditions such as obstructions, perforations, or extensive adhesions dictate the time needed to carefully dissect and handle tissues in an inflamed and fragile environment. In our study of 60 patients, we focused on 15 emergency cases, with seven in the suture group and eight in the staple group. While staples may offer some advantages in reducing surgical time, the urgency and complexity of the underlying condition are crucial in determining the total duration of emergency surgeries. If more emergency cases were included in our study, a clearer pattern might emerge. Therefore, in our study, comparing staple use with surgery duration in emergency cases did not show statistical significance.

Comparing the time until the return of bowel sounds for the two groups

In our study involving 60 patients, among the elective surgeries (45 cases), we observed that the staples group showed a notably earlier return of bowel sounds, typically within the second to third POD. A p-value of 0.02 favored stapled anastomosis for early return of motility in elective cases. This advantage in the staple group can be attributed to reduced contamination of the peritoneal cavity during elective surgeries, minimal bowel handling during staple use, and earlier closure of the abdominal cavity. Conversely, the hand-sewn technique involves more handling of the bowel with instruments such as bowel clamps and atraumatic forceps, exposing the bowel to a longer period outside the body [7]. The literature also suggests that the number of layers in the anastomosis may influence bowel sound recovery, with single-layer techniques often resulting in earlier return than double-layer methods [14]. However, all the anastomoses were performed in two layers in our study. Nevertheless, one study found minimal difference between suturing and stapling in terms of bowel sound recovery [15]. In emergency cases, we found no significant difference in the return of bowel sounds between the staple and suture groups. This lack of distinction in the early return of bowel sounds in emergency surgeries, unlike elective cases, may be due to the nature of the emergency condition, which can lead to greater contamination of the peritoneal cavity and increased edema of the bowel and surrounding tissues.

Comparing the duration of stay in days post surgery for the two groups

In the staple group in the elective setting, only two patients were discharged after the seventh POD, whereas in the suture group, eight patients experienced delays beyond the seventh day. The p-value of 0.04 indicates a significant difference favoring staple use in early patient discharge, possibly due to faster return of bowel sounds and shorter surgical duration, which reduces postoperative stress. Published research supports these findings of faster recovery and discharge in staple-assisted surgeries [15]. Conversely, among emergency cases, we found no significant difference in hospital stay duration between the suture and staple groups. Patients in both groups experienced prolonged stays due to delayed bowel sounds, considering the underlying pathology and complications related to the anastomosis site. Some original articles claim that staple use has some advantages regarding early discharge and early return of bowel sounds, even in emergency cases [8]. These findings from our study suggest that while staple use may expedite recovery in planned surgeries, it may not have the same impact in emergencies where other factors may influence hospital stay durations.

Comparing anastomotic leaks in the two groups

In every instance, the presence of anastomotic leakage on the first or second POD is a consequence of technical factors, and it is common for anastomotic leakage, which is caused by interference in the natural healing mechanism, to manifest near the end of the first week immediately following surgery [1, 3].

In our study, we observed a significantly lower rate of anastomotic leaks in the staple group during elective surgeries compared to the suture group. This advantage may be attributed to the additional seromuscular reinforcing sutures typically applied after using a linear cutter stapler or, in cases where a triple-layer endo GIA stapler is used, this inherently adding a third layer without requiring additional sutures [9]. The overall anastomotic configuration is a functional end-to-end type, which could contribute to this lower leak rate [16-18]. Additionally, maintaining normal albumin levels before surgery may also play a role, as lower albumin levels are associated with higher leak risks. However, some studies suggest that there is no significant difference in leak rates between suture and staple groups in elective cases [13, 19, 20]. The mechanism of surgical staplers is designed to evenly distribute pressure across the intestinal wall along the entire staple line, ensuring uniform staple thickness throughout the anastomosis. Continuous advances and modifications of surgical staplers by various medical companies highlight the importance of surgeons understanding key principles such as compression, staple height, tissue thickness, compressibility, and suitable tissue types for staple application [21]. Conversely, the higher rate of anastomotic leaks for elective cases in the suture group may be influenced by the surgeon’s technique and experience in performing the anastomosis. The configuration type of anastomosis, whether side-to-side, end-to-end, or end-to-side [22], also plays a role, with end-to-end anastomoses generally associated with higher leak rates, particularly when the mesentery close to the transacted end has a precarious blood supply [23]. However, some articles state that end-to-end is better in the long term when comparing the reintervention rates of the other configurations [24].

In emergency cases, no significant difference existed between the suture and staple groups regarding leak rates. However, it is notable that among the 15 emergency surgeries in our study, eight patients underwent stapled anastomosis, with three experiencing leaks. Although some articles state that no benefit exists in diversion compared to end ileostomy [25], in our cases, two patients underwent diversion ileostomy, and one was managed with pigtail drainage around the anastomosis site. Among the seven emergency cases where patients underwent suture anastomosis, only one had a leak, which was managed conservatively using antibiotics and ultrasound-guided drainage. These findings suggest a higher arbitrary leak rate with staples in emergency cases compared to sutures. This could be attributed to factors such as low albumin levels, increased bowel wall edema, poor tissue perfusion status, poor intestinal microbiome composition, and the contaminated peritoneal environment typical in emergency settings. In conditions such as Crohn’s disease, where patients may be on long-term immune suppression, the probability of a leak is higher regardless of the technique or method of anastomosis used. Certain articles state that stapled side-to-side anastomosis is better in conditions such as Crohn’s [26].

Recent studies conducted in the past two to three years have explored the role of manual tremors transmitted during hand-operated stapler applications on staple line-related leaks and the anastomotic healing time. These studies have also investigated factors such as the pressure applied by the stapler, the distance between the two limbs of the stapler, and the time taken from compression initiation to firing, often using strain gauge and microprocessor technology [27-29]. The consensus from these studies is that battery-powered automated stapling devices tend to yield better outcomes and lower leak rates than manual staplers.

Comparing the albumin level to the leak rate within the suture and staple groups

In our study, in the suture and staple groups, the anastomotic leak rate was significantly dependent on the albumin level (p-value of 0.001 in the suture group and 0.0006 in the staple group). We considered albumin below 3 mg/dl as low. However, almost all the elective cases had an albumin level above 3 mg/dl except one patient with low albumin in the staple group, who experienced no leak, and two patients with low albumin in the suture group, who experienced leaks. Two other cases in the suture group had leaks without low albumin. In the emergency setting, almost all the patients with low albumin had leaks in the suture and staple groups. This association of leaks in patients with low albumin levels is well-established by various research articles [30, 31]. These findings highlight the clinical relevance of monitoring albumin levels preoperatively as a potential predictor for postoperative complications such as anastomotic leaks, emphasizing the importance of nutritional status in surgical outcomes across different clinical settings.

Albumin, a protein essential for maintaining oncotic pressure, is critical in fluid balance, which affects bowel edema and the integrity of sutures or staples. Low albumin levels contribute to an increased risk of anastomotic disruption [32, 33]. Additionally, in emergency cases, the presence of fecal or bacterial contamination creates a challenging local environment that predisposes to anastomotic dehiscence.

Systemic inflammatory responses further hinder tissue healing processes essential for anastomotic recovery [34]. Apart from albumin, factors such as hemoglobin levels, C-reactive protein levels, and inotropic support also play crucial roles in facilitating complication-free anastomotic healing [35]. Overall, in our study, of the eight patients noted to have leaks, only two required loop ileostomy; the remainder were managed by nil orally, pigtail insertion for drainage, and intravenous antibiotics. Thus, more than half of the patients were managed without reoperation (diversion ileostomy); this scenario was published in an article stating that leaks occur late and do not frequently require a second surgery [36].

Comparing surgical site infections and pain scores of the two groups post surgery

For elective cases, we observed a significantly lower incidence of superficial SSIs in the staple group compared to the suture group. This reduction in SSI rates with staple use can be attributed to several factors. Staples involve less operation time and potentially less contamination of the surgical field, especially when inserting the stapler through a small enterotomy, compared to the prolonged exposure of the entire lumen during the initial layers of anastomosis of the suture technique. Our findings contrast with various published studies and meta-analyses that generally show no substantial difference in SSI rates between suture and staple techniques [37]. Additionally, in our study, no significant difference existed in SSI rates between the suture and staple groups in emergency cases. This suggests that using staples may decrease the risk of SSI in planned surgeries; however, this advantage may not extend to emergency surgical scenarios where other factors could influence infection rates.

Our study found that patients did not report a noticeable difference in pain between the two groups. This aligns with findings from other articles that suggest that pain after surgery is influenced by factors beyond the type of anastomosis. The overall condition being treated and the extent of the surgical procedure appear to have a greater impact on how much pain patients experience. Thus, pain levels can vary widely based on the specific health issue being addressed and the complexity of the surgery performed [38].

Limitations of the study

A limiting factor in our study is that we compared variables for the suture and staple groups for elective and emergency cases overall but did not compare outcomes for individual case types. Instead, elective and emergency cases were grouped separately (non-randomized). Larger studies are needed to compare outcome variables on a case-by-case basis in future research.

## Conclusions

In summary, based on our study, stapled anastomosis in elective surgeries offers several advantages over suture anastomosis, including shorter surgery durations, earlier return of bowel function, faster hospital discharge, and reduced risk of anastomotic leaks. Furthermore, staple use showed similar outcomes to sutures in terms of albumin levels, pain scores, and superior prevention of surgical site infections. All outcome variables in our study were evaluated during the initial 10 days post surgery. However, the long-term effects of sutured and stapled anastomosis, especially regarding rates of long-term anastomotic strictures, require further investigation.
